# Identification of acacia gum fermenting bacteria from pooled human feces using anaerobic enrichment culture

**DOI:** 10.3389/fmicb.2023.1245042

**Published:** 2023-10-10

**Authors:** Muhamad Hanif Rawi, Hui Yan Tan, Shahrul Razid Sarbini

**Affiliations:** ^1^Innovative Food Processing and Ingredients Research Group, Faculty of Food Science and Nutrition, Universiti Malaysia Sabah, Kota Kinabalu, Sabah, Malaysia; ^2^Department of Crop Science, Faculty of Agricultural Science and Forestry, Universiti Putra Malaysia Bintulu Campus, Bintulu, Sarawak, Malaysia; ^3^Halal Products Research Institute, Universiti Putra Malaysia, Serdang, Selangor, Malaysia

**Keywords:** gum arabic, *Acacia senegal*, gut microbiota, *Escherichia*, isolation, metabolism, butyrate

## Abstract

Commercial acacia gum (AG) used in this study is a premium-grade free-flowing powder. It is a gummy exudate composed of arabinogalactan branched polysaccharide, a biopolymer of arabinose and galactose. Also known as food additive, acacia gum (E414), which is presently marketed as a functional dietary fiber to improve overall human gut health. The health effects may be related to the luminal pH regulation from the short-chain fatty acids (SCFA) production. Studies suggested that amylolytic and butyrogenic pathways are the major factors determining the SCFA outcome of AG in the lower gut. However, the primary bacteria involved in the fermentation have not been studied. This study aimed to investigate the putative primary degraders of acacia gum in the gut ecosystem. Isolation and identification of gum-fermenting bacteria were performed through enrichment culture fermentation. The experiment was conducted in an anaerobic chamber for 144 h in three stages. The study was conducted in triplicate using an anaerobic chamber system. This culture system allows specific responses to support only bacteria that are responsible for gum fermentation among the gut microbiota. Five bacterial strains were isolated and found to be gum-fermenting bacteria. Based on the 16s RNA sequence, the isolates matched to butyrate-producing *Escherichia fergusonii*, ATCC 35469.

## 1. Introduction

Despite the development of culture techniques and fast-moving molecular identification technology, there is still a substantial amplitude of uncultured microbial diversity within the gut ecosystem, a vast number of gut microbes yet to be characterized (Lu et al., 2014; Shin et al., 2019). As reviewed by Rawi et al. (2020), human gut bacteria largely classified into *Bacteroides, Prevotella*, and *Ruminococcus*, which predominantly inhabit by phyla *Firmicutes* and *Bacteroidetes* (Walker et al., 2011). Another recent metagenomic analysis conducted by Forster et al. (2019) revealed that the most frequent genomes were *Ruminococcus bromii, Alistipes putredinis,* and *Eubacterium rectale*. All are known to colonize the human gut (Rajilić-Stojanović and deVos, 2014), confirming that these species are common members of the intestinal microbiota. These data suggest that, despite being known colonizers of the intestinal microbiota, these clades still contain considerable uncultured diversity. Therefore, the detection of many uncultured species assigned to this genus may reflect the current taxonomic limitations rather than biological signals. A review also suggested that specific microbial strains are responsible for the metabolism of certain substrates. Giving the impression that the gut environment is similar to the ecosystem will make the microbiome relationship within the gut more sensible. It can be postulated that the gut bacteria can be compared to the animal kingdom, while prebiotics or other indigestible substances in the colon serve as sources of food, similar to producers in an ecosystem. Just like in a food chain, a combination of food chains makes up a food web. The various undefined linkages between gut bacteria can be explained by their cross-feeding situation, which leads to both competitive and cooperative relationships through the intermediate fermentation products from one or more bacterial species to another. In the animal kingdom, there is a hierarchy of creatures, where prey and predators are further differentiated, and the prey typically includes primary to tertiary consumers. On the other hand, the bacteria could be categorized either as commensal, pathogenic or beneficial species. If an imbalance exists in the ecosystem, the condition of the colon is called gut dysbiosis (DeGruttola et al., 2016).

Enrichment culture is a closed system that can isolate organisms that utilize a specific nutrient substrate among complex microbial communities (Kishimoto et al., 2006). The enrichment culture employed a liquid growth medium containing acacia gum, creating an environment strictly desirable to isolating the primary substrate-utilizing organisms. This approach facilitates the isolation and subsequent study of the targeted microbes. Anaerobic enrichment culture techniques, as described by Beck (1971), are effective in isolating a variety of obligate and facultative anaerobic bacteria. The biological phenomena of the isolated bacteria were observed using enrichment culture techniques. The essence of this technique is to provide growth conditions that are favorable for the organism of interest and as unfavorable as possible for competing organisms. Simultaneously, an enrichment culture can be applied by modifying the media.

At least two decades of gut research have passed, and many of the unknowns before scientists now become clearer. Together with the host immune system, the gut microbiota acts as a barrier and prevents the invasion of pathogenic bacteria in the gastrointestinal tract. FOS has been extensively studied as a prebiotic that regulates and selectively stimulates bacterial populations in the colon (Tuohy et al., 2001; Palframan et al., 2002; Hidalgo et al., 2012; Cueva et al., 2013). Fermentation of AG results in bacterial proliferation (May et al., 1994). Thus, an increased population of colonic bacteria promotes the production of beneficial metabolites, such as short-chain fatty acids (SCFA), which play an important role in many physiological effects. The study involved observations on selective agar medium, with culture-dependent techniques dominating assessment methods during that period. Thus, selective media that only targeted beneficial bacteria were not sufficient to verify the fermentability of AG by colonic bacteria. In addition, AG been reported to break down in the colon of rats and humans (Ross et al., 1984; Walter et al., 1988; Phillips, 1998). While these studies were evaluated *in vivo*, there is no direct proof to support the findings since the parameters were only evaluated after the administration of the candidate after being fed AG during the study. In 2006, an animal study by Kishimoto et al. (2006) found that the predominant microbes from pooled cecal inocula of pigs responsible for AG fermentation and contributing to propionate production were *Prevotella ruminicola*-like bacteria. In contrast, studies using human and porcine fecal inoculum with 2% acacia gum isolated *Bifidobacterium longum* and *Bacteroides ovatus, Bacteroides oris, Bacteroides buccae,* and *Prevotella ruminicola*-like bacteria (Wyatt et al., 1986). While the study utilized human fecal samples, the conclusions drawn were primarily derived from culture plating techniques rather than molecular-based DNA sequencing. Notably, the latter method, which emerged in the 1970s, might not have been accessible in their laboratory during that period. Therefore, the traditional detection of many uncultured species assigned to this genus may reflect the current taxonomic limitations rather than biological signals. The chemical composition of AG varies slightly based on origin, sources, climate, season, tree age, and species (Williams and Phillips, 2009). The high carbohydrate content within these complex polysaccharides serves as the basis for evaluating the prebiotic potential of acacia gum. Species differences in AG were considered to determine the extent to which the species affected the fermentation performance of the colon. Thus, the evaluation of SCFA production and bacterial composition changes due to the input of AG as a substrate is the foundation for the benefits of human colon health. The limitation of simulating the interaction between the host and gut microbes in a fermentation system may also be one of the criteria for a fermentation system.

In this study, the enrichment technique was applied to AG in nutrient medium. A microbial strain that specifically utilized acacia gum as a nutrient source was isolated using an anaerobic enrichment culture fermentation system. Therefore, this study addressed possible pathways of AG fermentation by colonic bacteria. Investigation of the putative primary degrader of acacia gum based on enrichment culture was used to generate more information on the relationships between different microbial species in the gut. Hence, through this study, it is possible to enhance our understanding of the prebiotic applications of acacia gum in various industries, particularly nutraceuticals. Despite this, the current study is the first to show new insights towards the old knowledge about the prebiotic potential of AG. This study also opens the new perspectives on substrate-species relationship in the metabolism of food products.

## 2. Materials and methods

### 2.1. Acacia gum (AG) as the substrate

In this study, *Acacia senegal* gum (obtained from Natural Prebiotic Sdn. Bhd., Malaysia), which is a water-soluble, free-flowing powder. This commercially available product, marketed as a dietary fiber drink, consists of *A. senegal* gum in its raw form. Notably, the highly branched structure of this gum, composed of β-(1,3)-galactopyranose chains forming the backbone with branched side chains linked through the 1,6 positions comprising galactose, arabinose, rhamnose, and glucuronic acid, renders it resistant to human gut hydrolysis. These complex structures are not available as free sugar molecules for bacteria.

### 2.2. Anaerobic enrichment culture

The following methods was based on the study of Kishimoto et al. (2006) with modification. The experiment was conducted in a Bactronez-2 (*Sheldon* Lab, United States) anaerobic chamber following the manufacturer’s operating procedure. Fecal inoculum was obtained from three healthy human volunteers who fulfilled all the exclusion criteria as described previously. A portion of fecal material (10 g) from healthy volunteers was pooled and homogenized in a stomacher with buffer (1:10 w/v, pH 6.5, 1X PBS) at a normal speed for 2 min. An amount of 40 mL pre-reduced basal medium (pH 6.8) consisting of (peptone water (2 g/1), yeast extract (2 g/1), NaCl (0.1 g/1), K_2_HPO_4_ (0.04 g/1), KH_2_PO_4_ (0.04 g/1), NaHCO_3_ (2 g/1), MgSO_4_•7H_2_O (0.01 g/L), CaC1_2_•6H_2_O (0.01 g/1), tween 80 (2 mL/1), hemin (50 mg/1), vitamin Kl (10 μL), L-cysteine HCl (0.5 g/1), bile salts (0.5 g/1), resazurin (0.25 g/L) (4.0 mL) with 1% AG was added into 100 mL Schott bottles and were autoclaved before transferred into the anaerobic chamber.

The 10 mL of fecal slurry prepared was inoculated into the (40 mL) medium and placed in an incubator at 37°C and subjected to continuous stirring. Three independent vessels were run in parallel and served as replicates. An additional set of vessels containing only media, without AG (as negative control), is concurrently run alongside the treatments to verify and validate culture growth. An amount of 1 mL of the culture was sampled from each vessel at 0 (start of incubation), 6, 12, 18, 24, 30, 36, and 48 h after the beginning of incubation. At the end of the culture period (48 h), 10 mL of the culture was transferred to fresh medium, and the culture and sampling were repeated. At the end of the second incubation (48 h), 10 mL of the culture was transferred to fresh medium, and culture and sampling were repeated for the last stage.

### 2.3. Chemical and molecular ecological analyzes

The sampled culture (1 mL) was centrifuged at high speed of 13,000 rpm for 10 min. The resulting supernatant was used for SCFA analysis using ion-exclusion HPLC, as described previously. Bacterial genomic DNA was extracted using the GF-1 Bacterial DNA Extraction Kit (*Vivantis*) following the manufacturer’s protocol. First, 1 mL of the sample was centrifuged at 6000 × g for 2 min. The supernatant was completely removed, and the pellet was resuspended in 100 μL of Buffer R1 and aspirated with a pipette. The sample was treated with 10 μL lysozyme (50 mg/mL) and incubated at 37°C for 20 min. The cell suspension was centrifuged at 10000 × g for 3 min. The supernatant was completely decanted. The pellet was resuspended in 180 μL Buffer R2 and 20 μL Proteinase K. The suspension was denatured at 65°C for 20 min with occasional mixing every 5 min. Bacterial genomic DNA buffer (two volumes) was added to each sample. The suspension was homogenized and incubated at 65°C for 10 min. The samples were precipitated using 200 μL of absolute ethanol. The samples were transferred to a spin column assembled in a clean collection tube. The sample was centrifuged at 10000 × g for 1 min. Wash buffer was added before the second centrifugation with the same parameters. The flow-through was discarded. The pellet was eluted with preheated elution buffer (50 μL) and centrifuged (10,000 × g for 1 min) in a clean microcentrifuge tube. The extracted DNA was stored at 4°C until further analysis.

### 2.4. Polymerase chain reaction

The universal primers 27F (5′AGAGTTTGATCMTGGCTCAG) and 1492R (5’GGTTACCTTGTTACGACTT) from the first Base IDT were used to amplify the 16S rRNA gene regions of the extracted DNA. Amplification was performed with XP Cycle (*BioER*) using the following cycling conditions:94°C for 5 min; 27 cycles of 94°C for 1 min, 51.3°C for 1 min, 72°C for 1 min, and 72°C for 10 min. Amplicons were purified by agarose (*First Base*) gel electrophoresis using a PCR Purification Kit (*Qiagen, QIAquick*) prior to DNA sequencing to identify the strain species. The DNA ladder used was 100 bp plus (*Vivantis*) and pre-stained with 6X Loading Dye (*Vivantis*).

### 2.5. Organic acids analysis

The organic acid quantification was based on Rawi et al. (2021). The fermentation sample from each sampling period was pipetted into a 2 mL microcentrifuge tube for centrifugation (Centrifuge-5804*, Eppendorf*) at 13000 rpm for 10 min to obtain a clear supernatant. The supernatant was filtered through a 0.22 μm syringe filter unit (*Millipore*) into an HPLC vial (*Agilent Technologies,* Cheshire, United Kingdom). Prominence Series Liquid Chromatography (*Shimadzu Corp.,* Japan) using a reverse phase ion-exclusion C12 column (Rezex ROA, *Phenomenex*) was used for SCFA analysis. Analytes were detected using a UV detector at a wavelength of 210 nm. The isocratic mobile phase used was 0.25 mM sulphuric acid (H_2_SO_4_). A 15 μL sample was injected into the heated column (40°C) programmed to run in isocratic elution at a flow rate of 0.5 mL/min for 40 min. The peaks and response factors within the sample were calibrated and calculated using the LC Solutions software (*Shimadzu*). The standard solution contained of 12.5, 25, 50, 75, 100, 125, 150, 175, and 200 mM acetate, butyrate, propionate, and lactate.

### 2.6. Isolation of acacia gum fermenting bacteria

A portion (1 mL) of the culture was collected at the end of enrichment (144 h after commencement of the enrichment culture). This sample was subjected to serial 10-fold dilution (10^−2^–10^−6^) in anaerobic 1× PBS buffer. Diluted samples (100 μL) were plated on agar with the same constituents as the basal media, with the addition of AG and bacteriological agar (20.0 g). Incubation condition was at 37°C for 48 h. The developed colonies were randomly isolated and subjected to colony purification before being transferred to a fresh slant medium containing the same ingredients. Successfully isolated colonies were transferred to broth medium containing the same ingredients, except for agar. After incubation at 37°C for 48 h, organic acids were analyzed, and cell morphology was examined. This step is important for verifying the ability of the isolate to ferment acacia gum. The 16S rRNA gene sequences of selected isolates were examined.

### 2.7. Gram staining

Gram staining was performed as previously described (Coico, 2006). A thin layer of the cells was smeared onto a glass slide using a sterilized loop inside a drop of distilled water. The sample was heated over a spirit lamp and dried to fix onto a slide. Cells were stained with crystal violet and incubated for 1 min. Subsequently, the slide was rinsed under a gentle stream of water for approximately 5 s. The slides were then shaken to remove excess water. Next, an iodine solution (iodine and potassium iodide) was added and incubated for 1 min. Subsequently, the slide was rinsed under a gentle stream of water for approximately 5 s. The slides were then shaken to remove excess water. Next, in a slightly tilted position on the slide, a few drops of ethyl alcohol were run through the sample until the last purple color ceased to wash away from the smear and rinse with a gentle stream of water for approximately 5 s. The slides were then shaken to remove excess water. Safranin was added to the smear and incubated for 1 min. Washed with a gentle stream of water for approximately 5 s and excess water is blotted around the ages of the smear with bibulous paper prior to microscopic observation.

## 3. Results

### 3.1. Anaerobic enrichment fermentation

Enrichment experiments of AG were conducted *in vitro,* using an anaerobic chamber to contain the culture. This study aimed to identify the first-hand microorganisms in human feces responsible for the fermentation of acacia gum. Furthermore, the putative isolates were verified for their ability to ferment acacia gum in monocultures. To the best of our knowledge, this is the first study on enrichment cultures using a complete anaerobic chamber. The benefit of using an anaerobic chamber is that it eliminates the possibility that the target bacteria may be exposed to perpetuate atmospheric oxygen during the transfer of the culture at any point. It is worth noting that any exposure to oxygen might reduce the chances or viability of anaerobic bacteria, especially strictly anaerobic ones. Therefore, keeping exposure to atmospheric air at a minimum, the probability of culturing bacteria that were once very difficult to grow *in vitro* was higher.

### 3.2. SCFA concentration from enrichment culture

**Figure 1:**
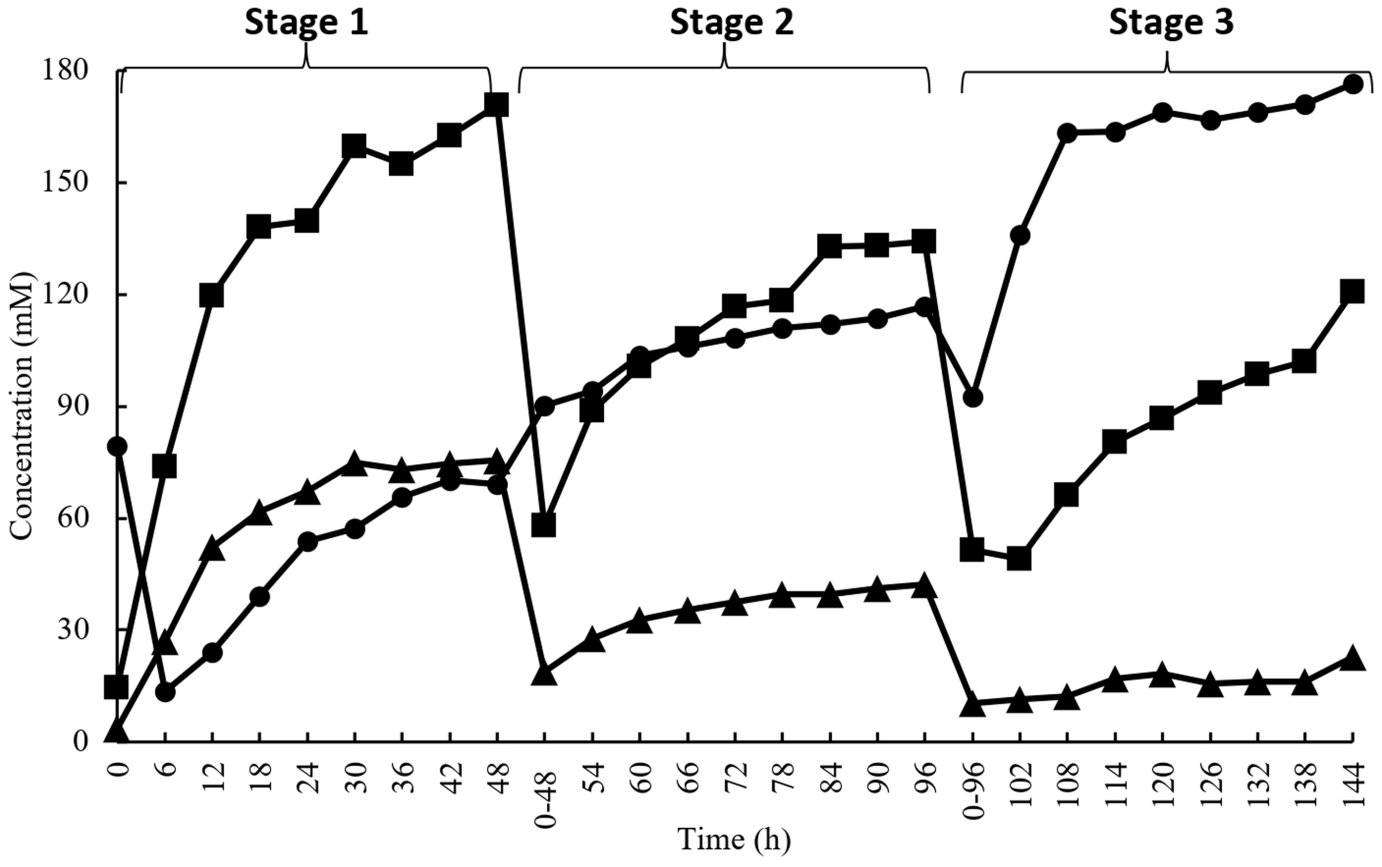
Short chain fatty acids concentration during enrichment culture on acacia gum (■, acetate; ▲, propionate; ●, butyrate). Transfer of culture to a fresh medium was performed at 48 h and 96 h.

Figure 1 shows different SCFA production during the 144 h of anaerobic enrichment by acacia gum. The enrichment culture consisted of three stages, where each stage indicates 48 h of incubation (stage 1:0–48 h, stage 2:48–96 h, and stage 3:96–144 h). The culture was transferred to fresh medium between stages (48 and 96 h). Acetate, propionate, and butyrate were the major SCFA produced during the incubation. At every stage, all the SCFA showed an incremental trend. The enrichment culture of acacia gum started with acetate and shifted towards health-promoting butyrate later in the incubation period. Acetate had the highest concentration at most of the time points in stages 1 and 2, whereas butyrate was the most abundant in stage 3. While the rate of acetate production decreased, that of butyrate production increased as enrichment progressed to stages 2 and 3. The molar proportion of propionate was very low and progressively diminished following the first and second medium transfer (Figure 1). A reduction in SCFA production was observed at every initial in each stage (0, 0–48, and 0–96 h). This can be expected from a multistage enrichment culture because the transferred culture to the subsequent stage contains a lower number of bacterial species. Therefore, lower bacterial compositions were present in the diluted samples transferred to the next series.

### 3.3. Monoculture fermentation of acacia gum by isolated strains

**Figure 2:**
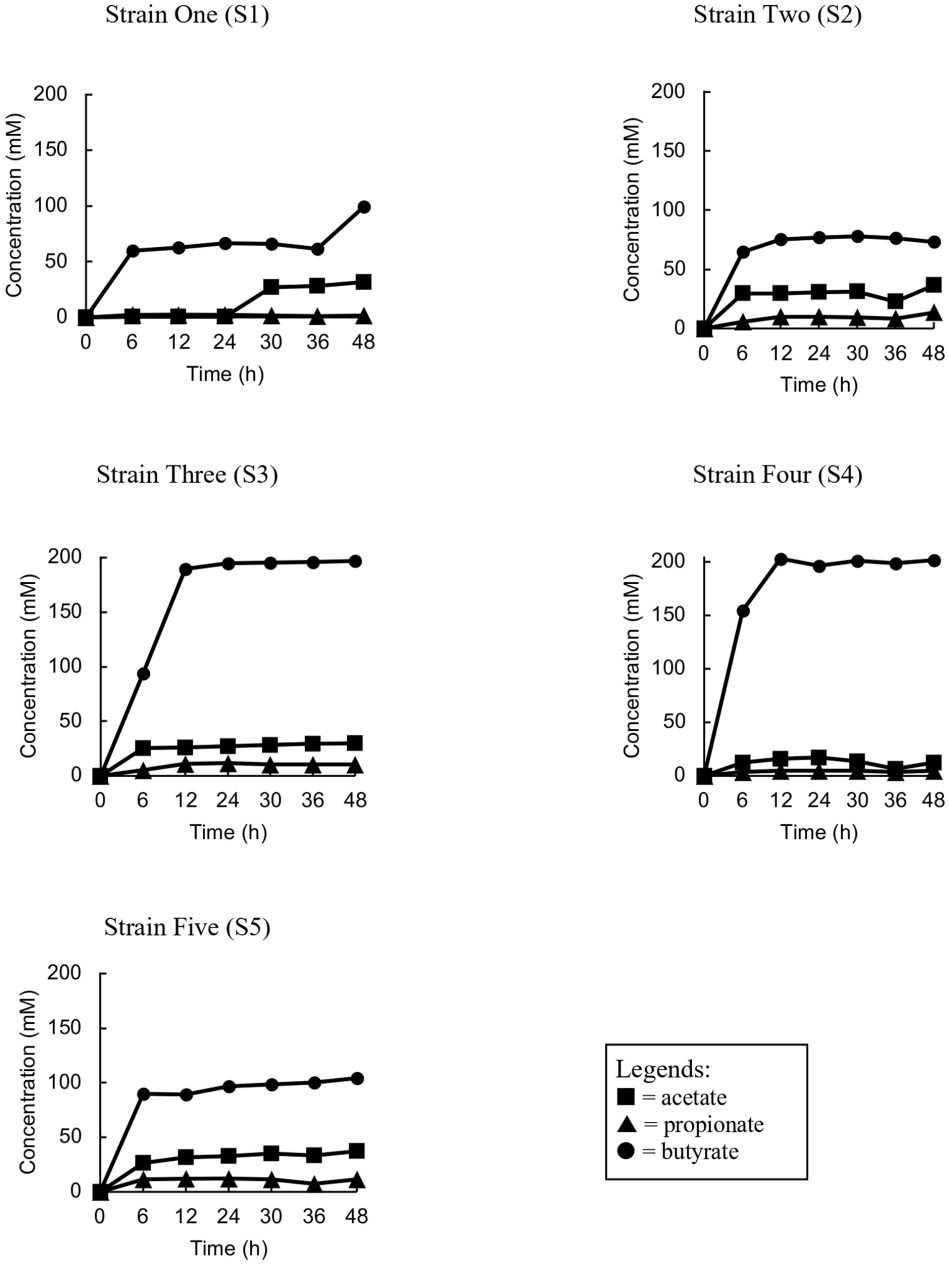
Trend analyses of the concentration of short chain fatty acids of each strain (S1, S2, S3, S4 and S5) during the monoculture fermentation.

The isolated bacteria were assessed to verify their ability to ferment acacia gum in pure culture. All five isolated strains with putative gum-fermenting abilities were reconfirmed by anaerobic monoculture fermentation for 48 h (Figure 2). All five isolates produced butyrate during monoculture, conforming to butyrate elevation in stage 3 of the enrichment culture, with a small amount of acetate and propionate. The elevation was significant as soon as 6 h for Strain 2, and 5 before it remained stationary at the same concentration level and continued until the end of fermentation. The same trend was observed for butyrate concentration at 6 h in strain 1, but it ended with a significant spike at 48 h. Furthermore, our observations, based on both culture-dependent methods and metabolite analysis, have confirmed that the isolated strains cannot thrive in basal media broth and agar without the presence of AG or other essential nutrients. This mirrors the observations made in mixed-culture feces, which also require the addition of nutrients for sustained growth. Therefore, two of the isolates (S3 and S4) were chosen to have the best fermenting ability (achieved maximum butyrate levels compared to other strains, 197 mM, and 203 mM, respectively).

### 3.4. Homology analysis of isolates

Five bacterial strains (S1, S2, S3, S4, and S5) were isolated at the end of the enrichment period. Among these bacteria, an evaluation was performed to identify different isolates based on their growth morphology. These isolates were found to be gram-negative. Based on the results of the BLASTN search (Table 1) of the 16 s ribosomal rRNA sequences of the five isolated strains, all isolates were matched (100% identity) to complete the sequence of the type of strain (holotype) *Escherichia fergusonii* ATCC 35469, partial sequence of *E. fergusonii* strain NRBC 102419, partial sequence of *E. coli* strain JCM 1649, and partial sequence of *Shigella flexneri* strain ATCC 29903.

**Table 1:** List of the sequences that showed similarity with the *Enterobacteriaceae* group.

16s rRNA gene	Strain	Accession no.	Sequence
*Escherichia fergusonii*	ATCC 35469	NR_074902.1	Complete
*Escherichia fergusonii*	NRBC 102419	NR_114079.1	Partial
*Escherichia coli*	JCM 1649	NR_112558.1	Partial
*Escherichia fergusonii*	ATCC 35469	NR_027549.1	Partial
*Shigella flexneri*	ATCC 29903	NR_026331.1	Partial

Values for the following parameters are identical for all sequences: Max Score and Total Score: 1664; Query cover: 100%; E value: 0.0; Identity: 100%.

## 4. Discussion

### 4.1. Substrate-species specific pathways

*Bifidobacterium* spp. is considered an important reference to see the impact of tested prebiotic since immense worked have been involving the use of species from this group and suggested a favorable impact belongs to the genera in the large intestine (Rawi et al., 2021). It is well known that gut fermentation of prebiotic within the colon produced metabolites known as short chain fatty acids (SCFA) beneficial to enhance the colonic health and reduce the risk of colonic diseases and disorders such as colonic cancer, inflammatory bowel disease and irritable bowel syndrome (Roediger, 1980; Jenkins et al., 1999; Floch and Hong-Curtiss, 2002; Hijova and Chmelarova, 2007). SCFA comprises of one to six carbon atoms organic fatty acids derived from anaerobic bacterial fermentation such as polysaccharide, oligosaccharide, or arabinogalactans as its reactant substrate (Miller and Wolin, 1979; Cummings and Macfarlane, 1991). According to Macfarlane and Gibson (1997), the ingested organic matter is digested through pathways such as glycolytic pathway and pentose phosphate pathway to transform into simple sugar molecules, pyruvate, energy for microbial growth and also other metabolites in the colon. From anaerobic fermentation of pyruvate, the process end up with a series of end products which include SCFA (Cummings, 1981). These SCFA are mainly metabolites such as acetate, propionate, butyrate, valerate and also other metabolites such as lactate, pyruvate, ethanol, succinate, gases of hydrogen, carbon dioxide and methane gas (Cummings, 1995; Levitt et al., 1995; Macfarlane and Macfarlane, 2003; Flint, 2006). The study of Macfarlane and Macfarlane (2003), Roberfroid (2005), and Cook and Sellin (1998) concluded that the production of SCFA is highly depending on the population of colonic microflora, gut transit time and also type of substrate. In other words, different sources of organic matter resulted in different composition of SCFA thus will affect colon physiology condition differently. The end fermentation products of AG include gases (methane, hydrogen, carbon dioxide) and short chain fatty acid (acetate, propionate, butyrate and valerate). Other intermediate products such as organic acids (lactate, succinate and formate), branch short chain fatty acid (BCFA) (isobutyrate and isovalerate), and alcohols (methanol and ethanol) are also produced with much smaller amount.

#### 4.1.1. Amylolytic bacteria

In the human colon, there is a range of undigested starch content, varying from 10% to a maximum of 40%. This undigested starch is fermented by colonic bacteria with amylolytic activity, resulting in the production of beneficial metabolites such as acetate, propionate, and butyrate (Salyers, 1979; Roediger, 1980; Cummings, 1981; Cummings and Macfarlane, 1991; Mortensen and Clausen, 1996). Amylolytic lactic acid bacteria (ALAB), have the ability to degrade starch using amylase during fermentation. These bacteria are naturally found in starchy fermented foods derived from cassava, sweet potatoes, grains, and maize. The breakdown of starch involves enzymes such as α-amylase for α-1,4 linkage, type I pullulanase for α-1,6 linkage, and amylopullulanases for both 1,4 and 1,6 linkages (Erra-Pujada et al., 1999). Species from three major phyla, namely *Firmicutes*, *Bacteroidetes*, and *Actinobacteria*, are known to participate in starch fermentation. *Lactobacillus* strains are generally considered amylolytic bacteria, but some *Bifidobacterium* strains isolated from the human gastrointestinal tract also exhibit amylolytic activity (Ji et al., 1992; Lee et al., 1997, 1999). Additionally, Crittenden et al. (2001) identified two amylolytic strains, *Bifidobacterium pseudolongum* (ATCC 25526) and *Bifidobacterium adolescentis* (VTT E-001561), which prefer α-1,4-linked glucose sugars. Undigested starchy substrates that survive upper digestion undergo various pathways in the colon, ultimately contributing to lactic acid production. Starch is initially broken down into simpler sugars through enzymatic saccharification, acid or alkali hydrolysis, and the action of amylolytic microbes. This is followed by a second-step fermentation by lactic acid bacteria (LAB) to produce lactic acid. Notably, amylolytic lactic acid bacteria (ALAB) have the unique ability to directly metabolize starch into lactic acid without the need for the first hydrolysis step. This pathway is also observed in co-culture simultaneous saccharification and fermentation, involving a mixture of LAB and ALAB or the integration of two or more fermentation steps. Certain bacteria, such as *Butyrivibrio fibrisolvens*, *Roseburia inulinviorans*, and *Roseburia intestinalis*, produce cell-associated amylase and are known as butyrate-producing bacteria. Other predominant amylolytic lactic acid bacteria (ALAB) include *Lactococcus*, *Streptococcus*, *Pediococcus*, *Carnobacterium*, *Bacteroides*, *Fusobacterium*, and *Butyrivibrio* (Macfarlane and Englyst, 1986; Bhanwar and Ganguli, 2014). *Bacteroides vulgatus*, one of the most abundant strains in the human colon, is an amylolytic gram-negative anaerobic bacteria. *Bifidobacteria* produce amylase extracellularly, while *Bacteroides* strains like *B. vulgatus* and *B. ovatus* produce cell-bound amylase (Ji et al., 1992; Degnan et al., 1997). This partial hydrolysis of starchy material is efficiently converted into lactic acid by amylolytic bacteria in a single step, enhancing the economic feasibility of the fermentation process (Sanoja et al., 2000; Fossi and Tavea, 2013).

#### 4.1.2. Butyrogenic bacteria

Butyrate, a beneficial short-chain fatty acid (SCFA) metabolite, is produced by various commensal gram-positive microbes in the human gut. The identification of these butyrogenic bacteria is an important area of research. Butyrate is typically generated through the fermentation of colonic microbiota. The XIVa cluster, particularly related to *Eubacterium rectale*, *Eubacterium ramulus*, and *Roseburia cecicola*, is the most abundant group (42%) within this cluster (Barcenilla et al., 2000). Among the major butyrogenic colonic microbes frequently detected in human feces are those from the Clostridial group, primarily clusters IV and XIVa of the Firmicutes phylum. *Faecalibacterium prausnitzii* and *Eubacterium rectale* are included in Cluster IV, while *Roseburia* spp., *Eubacterium* spp., *Anaerostipes caccae*, *Butyrivibrio fibrisolvens*, and *Coprococcus* spp. are significant butyrogenic bacterial strains within Clostridium Cluster XIVa. *Subdoligranulum variabile* and *Anaerotruncus colihominis* from Clostridium Cluster IV are also involved in butyrate production (Vital et al., 2014). These butyrogenic microbes produce butyrate by fermenting dietary fiber that survives upper gastrointestinal digestion. This process involves saccharolytic pathways related to carbohydrate metabolism and, to a lesser extent, proteolytic pathways associated with protein metabolism (Vital et al., 2014). Furthermore, butyrogenic bacteria exhibit specificity in fermenting unique types of dietary fiber (Moro et al., 2019).

**Figure 3:**
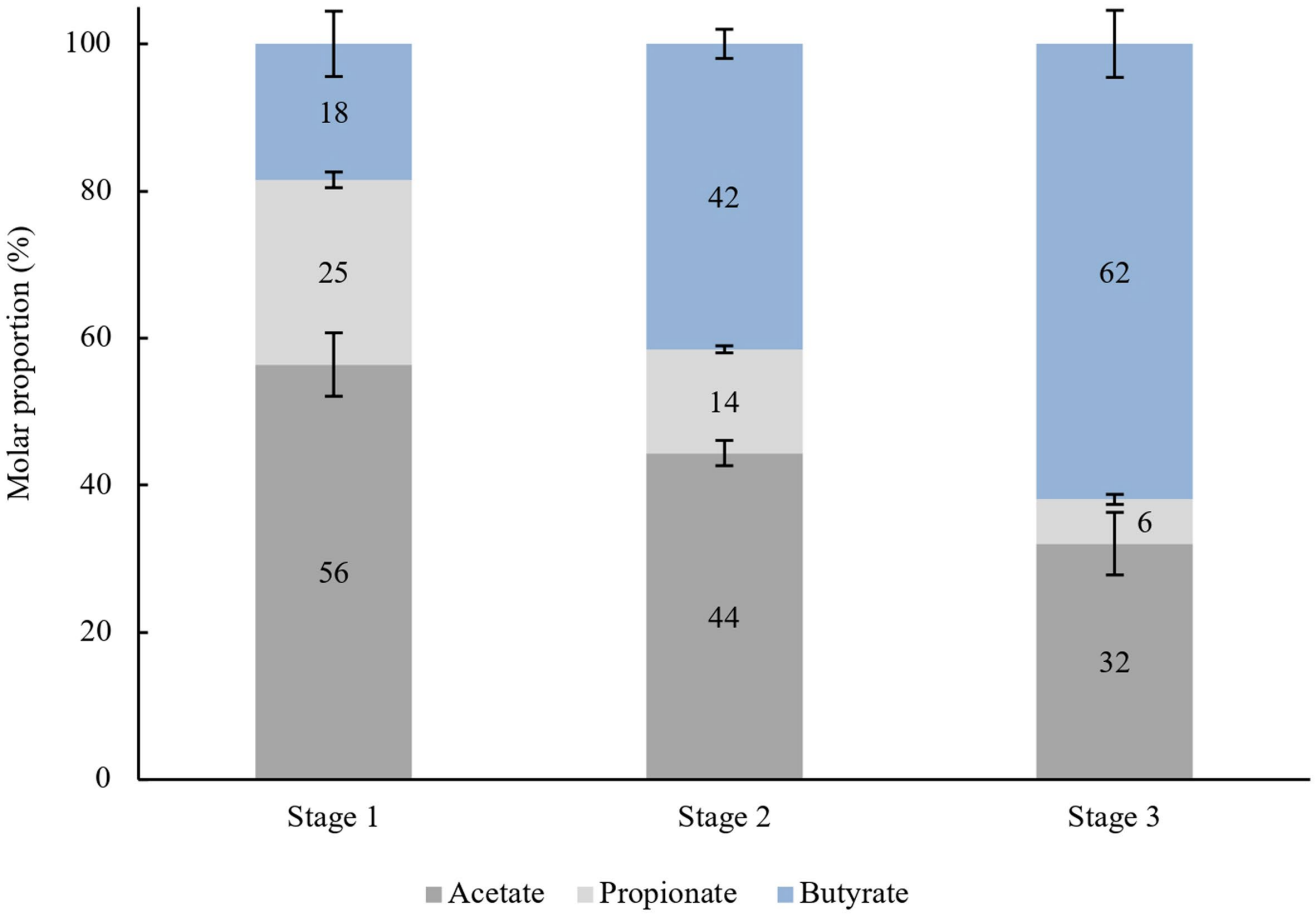
Average molar proportion of acetate, propionate and butyrate in every stage (48 h), *n* = 8.

Based on the results, in stage 1 (0–48 h) of the enrichment culture, the individual ratio of acetate:propionate:butyrate was 56:25:18, as shown in Figure 3, which is proportional to the ratio normally seen in the gut content. Different ratios were observed during stage 2 (48–96 h), and stage 3 (96–144 h) which are 44:14:42 and 32:6:62, respectively. Inevitably, a lower bacterial composition means lower production of SCFA compared to the previously cultured inoculum. This explains why the molar ratio of individual SCFA shifted to different proportions depending on which microorganisms were selected and could utilize acacia gum. As predicted, groups of amylolytic and butyrogenic bacteria were the major focus in determining the outcome of AG fermentation in the gut. Acetate, butyrate, and propionate exhibited an increasing trend from the beginning of every stage to the end of the same stage, but the gradient or the rate of production so to speak of acetate and propionate were significantly diminished compared to their respective times at the end of every stage (170 mM (48 h) > 134 mM (96 h) > 121 mM (144 h); 76 mM (48 h) > 42 mM (96 h) > 23 mM (144 h), whereas for butyrate, the opposite was observed, (69 mM (48 h) < 117 mM (96 h) < 176 mM (144 h). This explains why butyrate-producing bacteria were subsequently enriched, whereas others were screened out in the previous stage. Two independent pathways have been described for butyrate production. The less common pathway, the pyruvate from the Embden-Meyerhof-Parnas (EMP) pathway is further transformed by bacteria into three components: acetyl-CoA, succinate, and lactate. For acetyl-CoA, proceed into butyrate kinase pathways (Zhu et al., 2005) and are transformed into either butyrate or acetate with reciprocal transition. The acetate produced may be utilized by butyryl-CoA:acetate-CoA transferase as a CoA acceptor in another butyrate synthesis cycle carried out by *Roseburia intestinalis* (Duncan et al., 2002).

### 4.2. Acacia gum fermenting bacteria

Sequence analyzes of certain isolates often suggested that the 16 s rRNA sequence was from multiple strain as it is not easily distinguished. The sequence matched the complete sequence of *Escherichia fergusonii* ATCC 35469, whereas the rest only matched partial sequences available in GenBank for the Enterobacteriaceae group. They are most closely related to *Escherichia coli*. *E. fergusonii* was first isolated from the clinical specimens of a one-year-old boy by Farmer et al. (1985). Another study isolated *E. fergusonii* from healthy cattle (Balqis et al., 2018), and this type of strain was described as a non-pathogenic species (non-virulent in a mouse model) (Forgetta et al., 2012). Therefore, it is deemed commensal. The *E. fergusonii* strains tested positive for indole production, methyl red, lysine decarboxylase, ornithine decarboxylase, and motility. *E. fergusonii* can metabolize several compounds such as d-glucose, adonitol, L-arabinose, L-rhamnose, maltose, d-xylose, trehalose, cellobiose, and d-arabitol. However, they lacked the ability to ferment lactose, sucrose, myo-inositol, d-sorbitol, raffinose, and a-methyl-D-glucoside. *E. fergusonii* was found to have both α- and β-galactosidase (Maheux et al., 2014), encoded by LacZ (Reznikoff and Miller, 1978). β-galactosidase has three enzymatic activities (Huber et al., 1976). First, it degrades lactose into glucose and galactose prior to being forwarded into glycolysis. Next, the enzyme catalyzes the transgalactosylation of lactose to allolactose. The last function is to further break the allolactose into monosaccharides. In this perspective, β-galactosidase is best recognized for its high specificity for the galactose part β-D-galactopyranosides linked to a substituted indole substrate (Juers et al., 2012). Apart from this strain, other *E. fergusonii* species have been isolated from many diseases associated with animals, humans, and food samples (Wragg et al., 2009; Weiss et al., 2011; Maifreni et al., 2013).

Findings emerged with various statement of suggesting *E. fergusonii or E. coli*-related species as an opportunistic bacterium, and prone to be pathogenic in high number as reviewed by Gastra et al. (2014). It is thus interesting to mention that *Escherichia fergusonii* could be another anti-inflammatory commensal bacterium as evident in *Faecalibacterium prausnitzii* (also a major butyrate-producer), based on human clinical data (Sokol et al., 2009; Miquel et al., 2013). The lack of a selective medium for the isolation of *E. fergusonii* from materials such as feces, which have high background flora, has been a major hurdle to its detection. This is a novel study on the isolation of gum-fermenting *E. fergusonii* and the first to employ complete enrichment techniques in an anaerobic chamber. The potential role of *E. fergusonii* in biodegradation should also not be neglected (Sriram et al., 2011; Pasumarthi et al., 2013). In our interpretation, it is proposed that *E. fergusonii* encoded LacZ for β-galactosidase in membrane-associated proteins; thus, we speculated that extracellular hydrolysis occurred in the presence of acacia gum molecules. As mentioned in a gene insertion of *E. fergusonii* close relation, *Escherichia coli* mutant, it was revealed that there were resulting mutants that showed β-galactosidase activity in membrane fractions (Komeda, 1988). This is the first study to show that *Escherichia fergusonii* is a gum-fermenting bacterium. β-Galactosidase can act on β-D-galactopyranosides linked to oxygen via glycosidic bonds (Lederberg, 1950; Cohn and Monod, 1951), nitrogen (Sinnott and Withers, 1974), or sulfur and fluorine, but with significantly reduced catalytic efficiency (Wallenfels and Weil, 1972). Its active site specificity targeting d-galactose (Brockhaus et al., 1979; Nam Shin et al., 1980; Huber and Gaunt, 1983) and orientation (2, 3, and 4 positions) for reactions to activate is especially important because only D-galactopyranose, L-arabinopyranose, D-fucopyranose, and D-galactal react in the reverse direction when D-glucose is the other reactant. The enzyme can also slowly react upon exposure to p-nitrophenyl- a-L-arabinopyranoside and p-nitrophenyl-b-D-fucopyranoside (which do not have O_6_ hydroxyls) (Loeffler et al., 1979). All these studies conformed to the ability of *E. fergusonii* to break the complex chain of acacia gum polysaccharides in this study, considering the β-galactosidase specificity towards substrate structures. However, further studies are needed to understand the mechanism and capacity of *E. fergusonii* as an important commensal for fermenting complex polysaccharides.

### 4.3. Factors affecting the efficacy of *in vitro* fermentation

Previous research aimed at identifying acacia gum-fermenting bacteria found several different species and has not been consistent. Human and porcine fecal inocula with 2% acacia gum were used to isolate *Bifidobacterium longum*, *Bacteroides ovatus, Bacteroides oris, Bacteroides buccae,* and *Prevotella ruminicola*-like bacteria (Wyatt et al., 1986; Kishimoto et al., 2006). Kishimoto et al. (2006) reported that the isolated bacteria were propionate-producing bacteria. This highlights that propionate is the largest product of lactate metabolism (Stevani et al., 1991; Hove and Mortensen, 1995; Ushida and Hoshi, 2002). However, in our study, butyrate might have been derived from lactate as a precursor according to the monoculture isolates. Several factors can be ruled out to identify the differences between our study and the previous studies. *In vitro* fermentation systems are commonly used to simulate the human gastrointestinal environment for research purposes. To accurately mimic *in vivo* conditions, various factors need to be carefully controlled, including the type and concentration of substrate, preparation of inoculum, type, and components of the medium, incubation conditions, and stability of maintaining these conditions. The type and concentration of substrate used in prebiotic studies have a significant impact on the fermentation system. Different substrates react differently in the same system, and their physicochemical properties influence the availability of nutrients for bacterial growth and metabolism. Factors such as solubility, particle size, and nutritional composition of the substrates, as well as their structural properties, affect the accessibility of nutrients for bacteria. Soluble fiber is reported to have a higher influence on bacterial growth compared to insoluble fiber. Carbohydrate-type substrates lead to carbohydrate metabolism and the production of metabolites like lactate, acetate, butyrate, and propionate, while protein-type substrates contribute to protein metabolism and the production of branch chain fatty acids (BCFA).

The type and ratio of inoculum used in fermentation also affect the results. In general, inoculum can be saccharolytic (carrying out carbohydrate metabolism), proteolytic (carrying out protein metabolism), or a combination of both. Some bacteria can follow both pathways depending on substrate availability. Using a proteolytic inoculum for carbohydrate-based substrates can decrease the efficacy of fermentation conditions. It is also possible to use a mixture of various inoculum strains in an *in vitro* fermentation system. Overall, controlling the type and concentration of substrate, as well as the type and ratio of inoculum, is crucial for accurately simulating the human gastrointestinal environment in *in vitro* fermentation studies. Fundamentally, inoculum sources by Stevani et al. (1991), Ushida and Hoshi (2002) and by Kishimoto et al., 2006 were isolated from pigs, but in current study, feces were pooled from all healthy human volunteers. Next, different media preparations were used in all these studies. The composition was either from very simple media or a filtered fecal slurry, whereas this study used a more intricate medium, based on Gibson and Wang (1994), which was tested to support the growth of intestinal bacteria during incubation. Furthermore, the pH of the initial incubation is also an important issue to consider. Acidity can affect the regulation of lactate conversion by microbiota (Mackie and Gilchrist, 1979; Counotte and Prins, 1981). As such, more butyrate is produced at pH 5.8–6.0 as compared to pH 6.9 as lactate is converted into butyrate (Counotte et al., 1983). Butyrate production is linked to the transformation of other bacterial metabolites (Dominika et al., 2011). For example, *Megasphaera elsdenii* produces a wide range of butyrate from lactate, depending on the environmental pH (Counotte and Prins, 1981). Alternatively, the pH effect may be due to the tolerance of lactate-utilizing bacteria to acidity (Mackie and Gilchrist, 1979). In the current study, an initial pH of 6.8 in the enrichment culture was set and was not further controlled as the culture progressed. This explains how much acid level had progressed during the fermentation process, which may contribute to the regulation of butyrate-producing bacteria.

Next, the composition of microbiota in individuals could contribute to different SCFA profiles, despite all replications being administered under the same controlled conditions. Based on our findings, in the *in vitro* colon model, among all the replications, the human microbiota did not have the same SCFA proportion, suggesting that the microbiome metabolized lactate in a different manner. Additionally, the pooled feces in the enrichment study also showed a different outcome (butyrate vs. propionate) compared to the mix of the three human microbiota, which may suggest a propionate-producing type (Hove and Mortensen, 1995). Overall, the current study will serve as a basis for developing selection strategies for the isolation of new butyrate-producing bacteria from the human intestinal microbiota. In addition, there has been recent interest in determining the exact role of butyrate in ameliorating colonic diseases. It is also important to consider inter-individual differences in the study of prebiotics making progress into the market.

Furthermore, acacia gum fermentation showed that these food components are not digestible but are very rapidly fermented once in the large intestine, where fermentation transiently induces accumulation of lactate and therefore could greatly affect butyrate production based on our enrichment study. Acacia gum is a very heterogeneous, and many methods used to demonstrate such as in fractional precipitation, ion exchange chromatography (IEC), gel permeation chromatography (GPC), and hydrophobic affinity chromatography (HAC). Different species and compositions can exert different prebiotic effects. Acacia gum polysaccharides were fractionated to yield three main fractions: arabinogalactan, arabinogalactan protein, and glycoprotein (Randall et al., 1989). These fractions differ in their molecular weights and chemical compositions. Among these fractions, arabinogalactan represented 88% of the total weight and had a low molecular weight. The arabinogalactan moieties were not distributed evenly, and different compositions were quantified throughout the various molecular components present. Furthermore, acacia gum resisted enzyme hydrolysis, suggesting that the arabinogalactan component is enclosed in the core of the molecule and is inaccessible to the enzyme active sites. *A. senegal* possess the higher branching structure than *A. seyal* (78.2% vs. 59.2%) including the galactopyranoses, shorter arabinosyl side branches, and more rhamnopyranoses in terminal position could explained the degree of resistance of AG1 is better than AG2 according to the percent of total carbohydrate reduction (3.63% vs. 11.59%) (Rawi et al., 2021). Many studies agree that it is a highly branched structure composed of β-(1,3)-galactopyranose chains as the backbone with branched side chains joined through the 1,6 positions comprising galactose, arabinose, rhamnose, and glucuronic acid (Anderson et al., 1966; Churms et al., 1983).

Research on acacia gum as a potential prebiotic in this study confirmed other *in vitro* studies and complemented other *in vivo* studies involving animal and human trials. Undoubtedly that prebiotic research has opened a formerly unknown health potential, which is now becoming an alternative approach for mediating gut illness. For example, an animal study on obese mice by Everard et al. (2011) reported that prebiotic feeding improved glucose tolerance and reduced fat accumulation, oxidative stress, and inflammation. In a simulated model of the human colon, the use of synbiotic can alter the dominant bacteria and the production of short-chain fatty acids (SCFAs) in the fecal microbiota (van Zanten et al., 2012). Thus, there is a need to understand the role of gut microbiota in nutrition and health (Valdes et al., 2018).

## Data availability statement

The original contributions presented in the study are included in the article/supplementary materials, further inquiries can be directed to the corresponding author.

## Ethics statement

The studies involving humans were approved by Universiti Research Ethics Committee, Universiti Putra Malaysia (JKEUPM). The studies were conducted in accordance with the local legislation and institutional requirements. The participants provided their written informed consent to participate in this study.

## Author contributions

MHR, HYT, SRS contributed to conception and design of the study and wrote sections of the manuscript. MHR wrote the first draft of the manuscript. All authors contributed to the article and approved the submitted version.
